# *Cordyceps* inhibits ceramide biosynthesis and improves insulin resistance and hepatic steatosis

**DOI:** 10.1038/s41598-022-11219-3

**Published:** 2022-05-04

**Authors:** Ying Li, Chad Lamar Talbot, Bhawna Chandravanshi, Alec Ksiazek, Ayushi Sood, Kamrul Hasan Chowdhury, J. Alan Maschek, James Cox, Adhini Kuppuswamy Satheesh Babu, Henry A. Paz, Pon Velayutham Anandh Babu, David K. Meyerholz, Umesh D. Wankhade, William Holland, E. Shyong Tai, Scott A. Summers, Bhagirath Chaurasia

**Affiliations:** 1 Department of Nutrition and Integrative Physiology and the Diabetes and Metabolism Research Center, University of Utah, Salt Lake City, UT USA; 2 Department of Biochemistry, University of Utah, Salt Lake City, UT USA; 3 Department of Pathology, Carver College of Medicine, University of Iowa, Iowa City, IA USA; 4 Arkansas Children’s Nutrition Center, Little Rock, AR USA; 5 Department of Pediatrics, College of Medicine, University of Arkansas for Medical Sciences, Little Rock, AR USA; 6 Yong Loo Lin School of Medicine, National University of Singapore, Singapore, Singapore; 7 Saw Swee Hock School of Public Health, National University of Singapore, Singapore, Singapore; 8 Division of Endocrinology, Department of Internal Medicine, Carver College of Medicine, Fraternal Order of Eagles Diabetes Research Center, University of Iowa, Iowa City, Iowa City, IA 52242 USA

**Keywords:** Obesity, Type 2 diabetes, Metabolic syndrome

## Abstract

Ectopic ceramide accumulation in insulin-responsive tissues contributes to the development of obesity and impairs insulin sensitivity. Moreover, pharmacological inhibition of serine palmitoyl transferase (SPT), the first enzyme essential for ceramide biosynthesis using myriocin in rodents reduces body weight and improves insulin sensitivity and associated metabolic indices. Myriocin was originally extracted from fruiting bodies of the fungus *Isaria sinclairii* and has been found abundant in a number of closely related fungal species such as the *Cordyceps*. Myriocin is not approved for human use but extracts from *Cordyceps* are routinely consumed as part of traditional Chinese medication for the treatment of numerous diseases including diabetes. Herein, we screened commercially available extracts of *Cordyceps* currently being consumed by humans, to identify *Cordyceps* containing myriocin and test the efficacy of *Cordyceps* extract containing myriocin in obese mice to improve energy and glucose homeostasis. We demonstrate that commercially available *Cordyceps* contain variable amounts of myriocin and treatment of mice with a human equivalent dose of *Cordyceps* extract containing myriocin, reduces ceramide accrual, increases energy expenditure, prevents diet-induced obesity, improves glucose homeostasis and resolves hepatic steatosis. Mechanistically, these beneficial effects were due to increased adipose tissue browning/beiging, improved brown adipose tissue function and hepatic insulin sensitivity as well as alterations in the abundance of gut microbes such as *Clostridium* and *Bilophila*. Collectively, our data provide proof-of-principle that myriocin containing *Cordyceps* extract inhibit ceramide biosynthesis and attenuate metabolic impairments associated with obesity. Moreover, these studies identify commercially available *Cordyceps* as a readily available supplement to treat obesity and associated metabolic diseases.

## Introduction

Increased accumulation of lipid metabolite, ceramide is a key characteristic of obesity wherein it contributes to the impairments in glucose and lipid metabolism^1,2^. The synthesis of ceramides occur due to excess of saturated fats, wherein the incoming acyl-CoA couple with amino acid serine to produce sphingolipid, ceramide^3^. Ceramide synthesis is further fueled by inflammation and other stress stimuli associated with obesity^4,5^. Numerous studies using pharmacological inhibitors or genetically engineered mice reveal that inhibiting ceramide synthesis or stimulating ceramide degradation in rodents prevents or reverses diabetes, steatohepatitis, hypertension, cardiomyopathy, and atherosclerosis^6–16^. In addition, increasing number of recent studies have demonstrated a strong relationship between tissue and circulating ceramides and insulin resistance, hepatic steatosis, diabetes and cardiovascular diseases^17–26^. These findings suggest that targeting ceramide biosynthesis may offer a novel means to treat obesity and associated comorbidities.

Pharmacological agent myriocin, a selective and potent inhibitor of serine palmitoyl transferase (SPT), the first enzyme in the ceramide biosynthesis pathway has been a workhorse reagent demonstrating a potent efficacy in inhibiting ceramide synthesis and preventing as well as reversing obesity and associated diseases in rodents^7,9,15,27^. Myriocin was originally isolated from fruiting bodies of the fungus *Isaria sinclairii* (辛克莱棒束孢) and since then has also been found in a number of closely related fungal species such as the *Cordyceps*^28,29^*.* Despite having potent efficacy in rodents, purified myriocin is not an approved drug for human consumption. However, extracts from *Isaria sinclairii* and *Cordyceps sinesis* have been an essential component of traditional Chinese medication that has been consumed for thousands of years for the treatment of numerous indications, including diabetes^30–35^. *Cordyceps* gained worldwide prominence after 1993 following admittance from world record breaking Chinese long-distance runners that they consumed *Cordyceps* tonic during their training periods^30,32,33,36^. Since then, *Cordyceps* extracts have been increasingly sold as nutritional supplements in numerous countries, including United States and are being consumed by a large population globally. Numerous studies in rodents have found *Cordyceps* to reduce fat content, improve insulin sensitivity, hypertension and resolve hypertriglyceridemia and hypercholesterolemia^37–41^. In small clinical studies, the *Cordyceps* extract was found to ameliorate nephrotoxicity^42^. Moreover a follow-up study found that long-term-consumption of *Cordyceps* extracts for up to 3-years improved kidney function^43^. Importantly, genotoxic studies revealed that these extracts had no mutagenicity and is listed as one of the safest drug class (Class 1 drug) in the *American Herbal Products Association Botanical Safety Handbook*^44–46^. Despite, being safe and obvious therapeutic benefits of *Cordyceps* extracts, relatively nothing is known about the mechanisms via which *Cordyceps* exhibits these beneficial effects on energy and glucose metabolism. Hence, *Cordyceps* extracts are not approved for human consumption for the treatment of metabolic diseases.

Since the ceramide biosynthesis inhibitor myriocin is extracted from *Cordyceps*, we hypothesized that, myriocin might be the bioactive ingredient in *Cordyceps* and that ensuing reductions in ceramide biosynthesis might account for the beneficial effects of *Cordyceps* on energy and glucose metabolism. Screening commercially available *Cordyceps* extracts from Asia and the United States via mass spectrometry, we demonstrate that myriocin is indeed present in *Cordyceps* extracts but at variable concentrations*.* Moreover, treatment of mice with an obesogenic diet supplemented with myriocin containing *Cordyceps* at a dose equivalent to that currently being consumed by humans, reduces ceramide accumulation and improves energy homeostasis, insulin sensitivity and resolves hepatic steatosis with an alteration in the gut microbial ecology. Collectively, these studies provide proof-of-principle data in rodents supporting investigations on the therapeutic utility of commercially available myriocin containing *Cordyceps* to inhibit ceramide synthesis for the treatment of obesity, insulin resistance and hepatic steatosis.

## Results

### Identification of commercially available myriocin containing *Cordyceps* extracts

**Figure 1:**
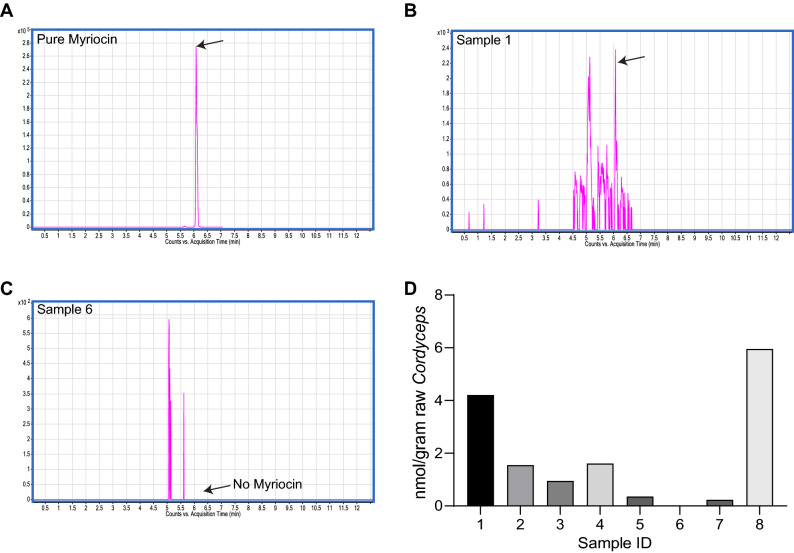
Screening of *Cordyceps* extracts for myriocin content via mass-spectrometry. (**A**) LC/MS chromatogram of purified myriocin and *Cordyceps* Sample 1 (**B**) and Sample 6 (**C**) depicting myriocin content. (**D**) Quantification of myriocin content in Samples 1–8 as determined by LC/MS.

There are over 750-species of *Cordyceps.* Among these, *Cordyceps sinesis* is the species that is most famous and classified as drugs in the Chinese Pharmacopeia^47^. Extracts isolated from these species of *Cordyceps* have been previously reported to vary in medicinal efficacy due to regions where these are cultivated and are increasingly in short supply due to difficulties in collecting natural *Cordyceps* that are grown at 3000–4000 meters above sea levels around the Himalayan mountains in Tibet and high regions of China^30,36^. The development of recent technologies that enable artificial cultivation and production of *Cordyceps* has enabled to overcome the supply limitations and to some degree the heterogeneity in *Cordyceps* composition, such that extracts from these species of *Cordyceps* are now available as supplemental medicinal capsules globally including via various E-commerce platforms^30,36^. Moreover, these artificially cultivated *Cordyceps* were found to possess similar potency to those grown in wild^48,49^. Here we investigated firstly, whether myriocin, a pharmacological inhibitor of serine palmitoyl transferase (SPT), the first enzyme in the ceramide synthesis pathway, that is present and, in some cases, isolated from *Cordyceps*^28,29^, is enriched in commercially available *Cordyceps* extracts approved for human consumption. Secondly, if myriocin is present in commercially available *Cordyceps* extracts, do different extracts contain equivalent amounts of myriocin. Therefore, we acquired eight different artificially grown *Cordyceps* extracts or wild type *Cordyceps* packaged as capsules or sold as wild mushrooms (Sample 1–8, Table 1) commercially available in United States and Asia (Singapore and China) and subjected them to mass-spectrometry analysis (Supplementary Fig. 1A). This analysis revealed that myriocin is indeed present in commercially available *Cordyceps* extracts and its content is highly variable in different *Cordyceps* preparations (Fig. 1A–D, Table 1 and Supplementary Fig. 1A–F). For example, sample 6 did not contain any traces of myriocin, sample 2–5 and 7 contained 0.5–2 nmol/g myriocin and sample 1 and 8 contained 4–6 nmol/g myriocin (Fig. 1A–D, Table 1 and Supplementary Fig. 1A–F).

**Table 1:** Table depicting the source, formulation and myriocin content in various *Cordyceps* extracts screened in this study.

Sample	Source, country of origin	Preparation	Myriocin content
1	Amazon.com, U.S.	Capsule	4–6 nmol/g
2	Amazon.com, U.S.	Capsule	0.5–2 nmol/g
3	Amazon.com, U.S.	Capsule	0.5–2 nmol/g
4	Amazon.com, U.S.	Capsule	0.5–2 nmol/g
5	Amazon.com, U.S.	Capsule	0.5–2 nmol/g
6	Eu Yan Sang, Singapore	Capsule	0
7	Eu Yan Sand, Singapore	Liquid	0.5–2 nmol/g
8	Qin Hai, China	Wild mushroom	4–6 nmol/g

### Treatment of mice with myriocin containing *Cordyceps* extract reduces serum ceramide content in obese mice and does not alter intestinal morphology

Treatment of mice with myriocin, inhibits ceramide biosynthesis and increases thermogenesis and energy expenditure, improves glucose homeostasis and resolves hepatic steatosis^27,50^. Therefore, we tested whether treatment of mice with *Cordyceps* extract containing myriocin at a dose that is recommended for human consumption resolves metabolic impairments associated with obesity. To this end, we sought to test the sample 1 since it is a capsulated *Cordyceps* extract that is artificially cultivated in United States, requires no further processing and is readily available for human consumption, whereas sample 8 that contained comparable amount of myriocin is a wild type *Cordyceps* acquired from China and needs further processing to prepare extracts.

**Figure 2:**
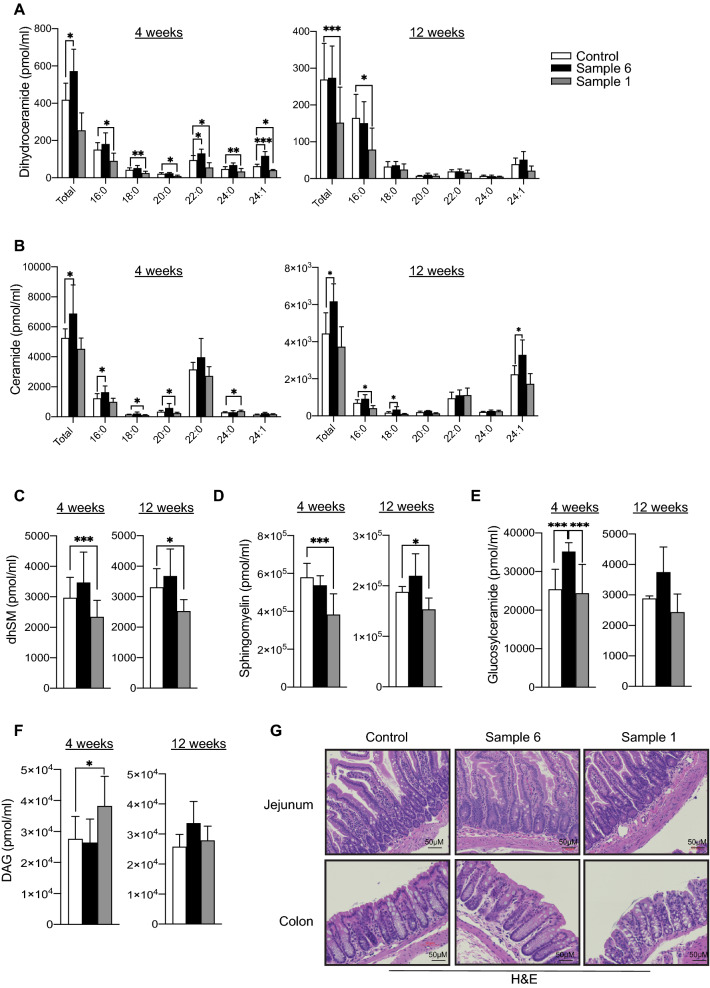
Treatment of mice with obesogenic diets supplemented with myriocin containing *Cordyceps* extracts reduces ceramide without altering intestinal morphology. Quantitative mass-spectrometry analyses of (**A**) Dihydroceramide, (**B**) Ceramide (**C**) Dihydro-sphingomyelin (dhSM), (**D**) Sphingomyelin, (**E**) Glucosylceramide and (**F**) Diacylglycerol content in serum isolated from C57Bl6/J fed high fat diets supplemented with starch (control), Sample 1 and sample 6 for 4-weeks and 12-weeks (N = 5 per group). (**G**) Following 12-weeks of dietary *Cordyceps* intervention small intestine was assessed histologically (H&E staining; N = 3 animals per group) and representative samples are shown. Values are expressed as mean ± SEM, *p < 0.05, **p < 0.001, ***p < 0.0001 vs control.

To test the efficacy of identified *Cordyceps* sample 1, in improving glucose homeostasis and nutrient metabolism, powdered sample 1 at a dose of 19.7 mg/kg was mixed into the high fat diet (HFD) as previously described^40^. This dose was calculated on the basis of pharmacological dose of sample 1 that was recommended by the manufacturer for human consumption. We also used a cohort with a higher dose (39.5 mg/kg) and observed intestinal inflammation, edema indicating intestinal toxicity at this dose and hence the study was discontinued. As controls we used high fat diet that either contained an equivalent amount of starch derived from rice or *Cordyceps* sample 6 that did not contain any traces of myriocin. Feeding 4-weeks old C57Bl6/J mice with HFD supplemented with sample 1 reduced circulating ceramides, dihydroceramides, dihydrosphingomyelin, sphingomyelin and glucosylceramides content as early as 4-weeks an effect that was sustained for the remaining 12-weeks of the treatment (Fig. 2A–E). In contrast sample 6 did not display any changes in serum ceramide levels compared to controls (Fig. 2A–E). Moreover, treatment with samples 1 but not sample 6 elevated diacylglycerol accumulation after 4-weeks but were not altered after 12-weeks (Fig. 2F). Taken together, these data suggest that sub-chronic and chronic treatment with HFD-supplemented with myriocin containing *Cordyceps* extract selectively inhibit synthesis of sphingolipids and its precursor ceramide.

Pharmacological and genetic inhibition of SPT presents with intestinal toxicity and alters intestinal morphology^51,52^. Therefore, we investigated whether treatment of mice with sample 1 *Cordyceps* extract had any impact on intestinal morphology. Histological analysis of the intestine following treatment with *Cordyceps* extracts from both sample 1 and sample 6 did not exhibit any alterations in gross intestinal morphology (Fig. 2G). Independent pathological assessment, further revealed no intestinal toxicity in animals treated with either sample 1 or sample 6 (Fig. 2G).

### Treatment of mice with myriocin containing *Cordyceps* increases energy expenditure in obese mice

**Figure 3:**
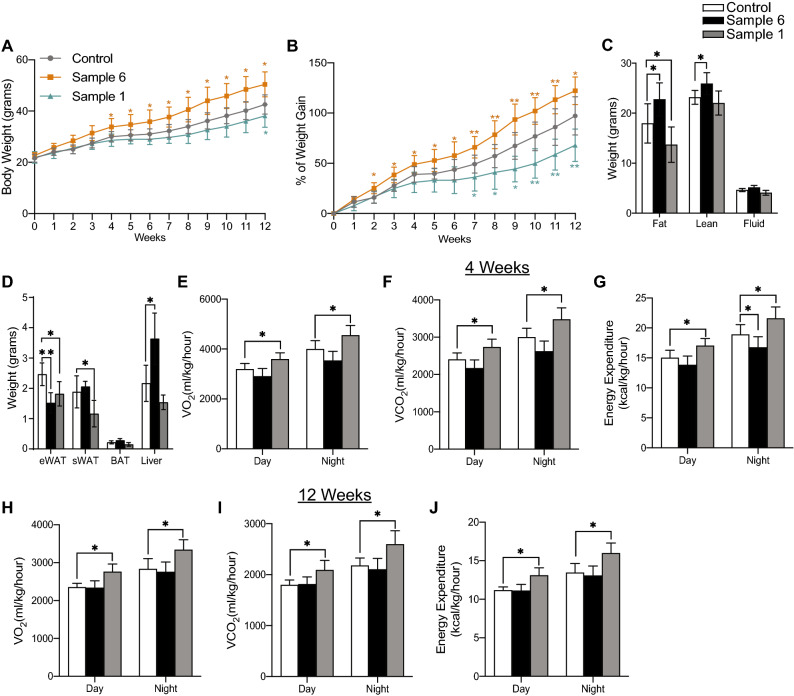
Treatment of mice with obesogenic diets supplemented with myriocin containing *Cordyceps* reduces body weight and increases energy expenditure. C57Bl6/J mice fed high fat diets supplemented with starch (control), Sample 1 and Sample 6 for 12-weeks. (**A**) Body mass and (**B**) Percentage weight gain was determined weekly. (**C**) Fat, lean and fluid mass were quantified by NMR and (**D**) organ weight was determined following euthanasia (N = 5–7 animals per group). Following 4-weeks (**E**–**G**) and 12-weeks (**H**–**J**) of dietary intervention supplemented with *Cordyceps* animals were placed in metabolic cages (CLAMS) from Columbus Instruments. During the subsequent 3-days, (**E**,**H**) oxygen consumption (VO_2_), (**F**,**I**) carbon dioxide production (VCO_2_) and (**G**,**J**) energy expenditure was quantified (N = 4–7 animals per group). Values are expressed as mean ± SEM, *p < 0.05, **p < 0.001, ***p < 0.0001 vs control.

Treatment of C57Bl6/J mice with HFD-supplemented with sample 1 reduced ~ 30% body weight (Fig. 3A,B) and altered lean/fat mass ratios (Fig. 3C), the phenotype that is consistent with our previous study following whole-body inhibition of SPT with the pharmacological agent myriocin^27^. In contrast, treatment with HFD-diet supplemented with sample 6, that lacks myriocin increased ~ 20% body weight and lean fat mass ratio (Fig. 3A–C). Consistent with reductions in fat mass observed, sample 1 treatment reduced weights of various adipose tissue depots such as the epididymal adipose tissue (eWAT) and the subcutaneous adipose tissue (sWAT) (Fig. 3D). In contrast treatment with sample 6 resulted in significantly heavier liver despite exhibiting reduced epididymal white adipose tissues (eWAT) weight (Fig. 3D). Treatment with sample 1 exhibited increases in oxygen consumption (VO_2_), carbon dioxide (VCO_2_) production, and energy expenditure without altering food intake (Fig. 3E–G) as early as following 4-weeks of *Cordyceps* treatment when the animals were equally obese. Interestingly, the increases in energy expenditure were sustained in sample 1 treated groups even after 12-weeks of HFD-treatment (Fig. 3H–J). No effect was observed on food intake, respiratory exchange ratio (RER), or locomotor activity following these interventions (Supplementary Fig. 2A–H). Surprisingly, Sample 6 that lacks myriocin did not alter any of these parameters after either 4-weeks or 12-weeks of treatment (Fig. 3H–J and Supplementary Fig. 2A–H).

### Myriocin containing *Cordyceps* alters adipose tissue morphology and increases adipose tissue thermogenic program

**Figure 4:**
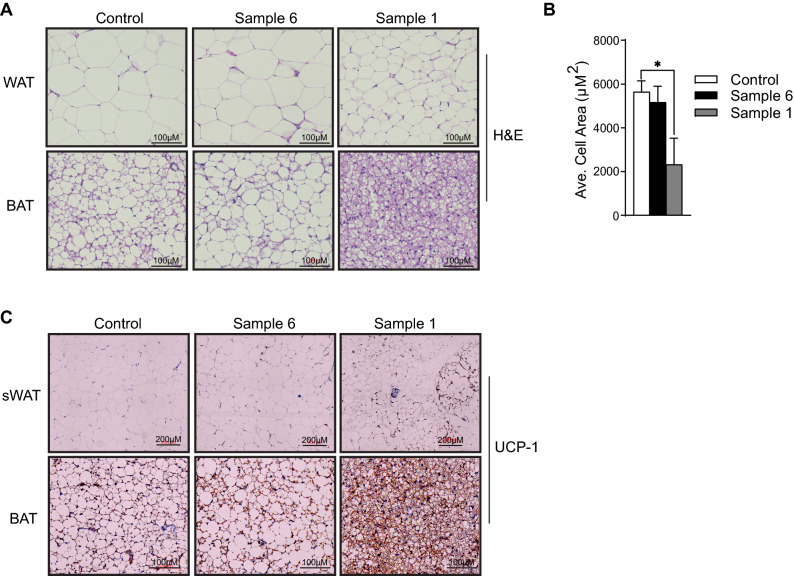
*Cordyceps* enriched in myriocin alters adipose tissue morphology and elevates adipose tissue thermogenic program. C57Bl6/J mice fed high fat diets supplemented with starch (control), Sample 1 and Sample 6 for 12-weeks. (**A**) Adipose tissue was evaluated histologically (H&E staining; N = 3–4 animals per group) following euthanasia and (**B**) images were evaluated to quantify adipocyte size. Following euthanasia, (**C**) sWAT and BAT depots obtained from Control, Sample 1 and Sample 6 treated mice for 12-weeks were evaluated by immunohistochemistry with antibodies recognizing UCP-1 (N = 4 animals per group). Values are expressed as mean ± SEM, *p < 0.05, **p < 0.001, ***p < 0.0001 vs control. *sWAT* subcutaneous fat pad, *BAT* Brown adipose tissue.

Histological assessment of different adipose tissue depots suggested that treatment with sample 1 produced a broad spectrum of effects on adipocyte morphology. Adipocyte cross-sectional area was significantly smaller (~ 50%) in sWAT and BAT depots indicating they are more metabolically active (Fig. 4A). By comparison, treatment with sample 6 exhibited increase in BAT adipocyte sectional area and had no changes in adipocyte size in the sWAT depot (Fig. 4B).

Our previous studies have identified that ceramides impair adipose tissue thermogenic program and conversely treatment of mice with myriocin increased adipose tissue thermogenic program and enhanced whole-body energy expenditure^27,50,53^. Therefore, we further determined if the improvement in energy expenditure following treatment with sample 1 increases adipose tissue thermogenesis. To this end, we performed immunohistochemistry for the thermogenic protein, uncoupled protein-1 (UCP1) in the sWAT and BAT. Consistent with the prior myriocin studies^27^, treatment with sample 1 induced UCP-1 expression in sWAT and BAT (Fig. 4C). Conversely, sample 6 treatment did not alter UCP-1 content in adipose tissues (Fig. 4C).

### Treatment of mice with myriocin containing *Cordyceps* improves insulin sensitivity and resolves hepatic steatosis in obese mice

**Figure 5:**
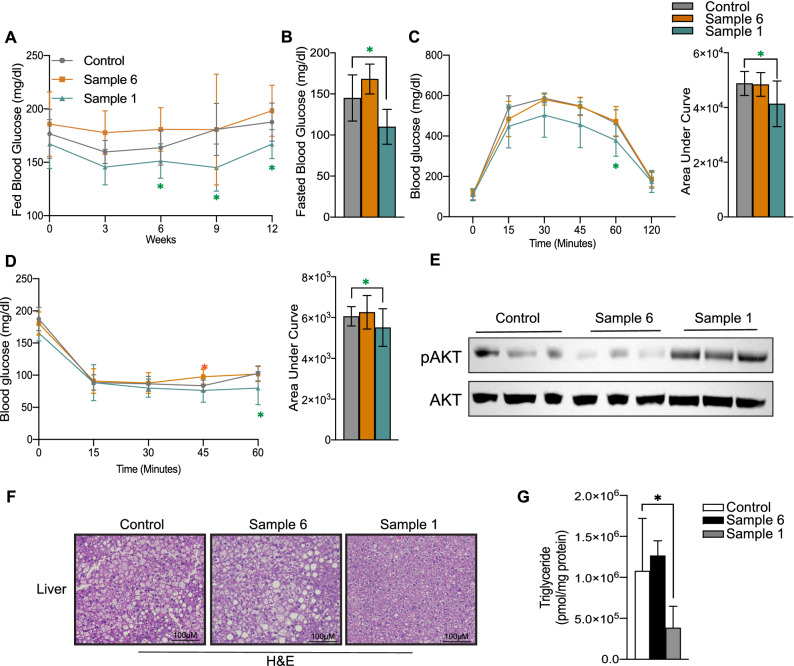
*Cordyceps* enriched in myriocin increases insulin sensitivity, resolves hepatic steatosis. C57Bl6/J mice fed high fat diets supplemented with starch (control), Sample 1 and Sample 6 for 12-weeks. (**A**) Fed blood glucose levels were determined every 3-weeks until the end of the study. (**B**) Fasted blood glucose levels were determined following 10-weeks of dietary intervention. (**C**) Glucose and (**D**) Insulin tolerance tests were performed following 4-weeks of dietary intervention (N = 5–7 animals per group). Following euthanasia, (**E**) liver pAKT (s473) and AKT was determined by Western Blotting (N = 3 animals per group), (**F**) livers were evaluated histologically (H&E staining; N = 3–4 animals per group) and (**G**) liver triglyceride content was determined by mass-spectrometry (N = 5 animals per group). Values are expressed as mean ± SEM, *p < 0.05, **p < 0.001, ***p < 0.0001 vs control.

We further assessed the efficacy of sample 1 in resolving metabolic impairments associated with obesity. Treatment of obese mice with sample 1, exhibited moderate improvements in random fed blood glucose as early as 3-weeks after the treatment, an effect that was sustained and significantly improved following 12-weeks of treatment (Fig. 5A). Importantly, sample 1 reduced fasted blood glucose following 12-weeks of treatment (Fig. 5B). Moreover, sample 1 treatment improved glucose tolerance (Fig. 5C) and insulin response during the insulin-tolerance test (Fig. 5D) without altering serum insulin content (Supplementary Fig. 2I). In contrast, sample 6 treatment did not affect fed and fasted blood glucose, exhibited no differences in glucose tolerance but impaired insulin tolerance, and exhibited elevated serum insulin levels (Fig. 5B,C and Supplementary Fig. 2I).

Ceramides impair systemic insulin sensitivity by inhibiting glucose uptake in peripheral tissues and compromising hepatic glucose suppression^2,54^. Moreover, treatment with myriocin, negates these metabolic effects^7,50,53^. We and other have previously found that these effects are mediated through the ceramides ability to inhibit insulin-stimulated Akt/PKB phosphorylation, an effect that is restored by inhibition of ceramide biosynthesis with myriocin^7,50,53–57^. Therefore, we further determined if the observed metabolic improvements in glucose metabolism caused by sample 1 were associated with improved signaling to Akt/PKB. Herein we found that consistent with prior myriocin studies^7,9,15^, treatment with sample 1 significantly enhanced hepatic Akt/PKB phosphorylation (Fig. 5E). In contrast, treatment with sample 6 did not have an effect on Akt/PKB phosphorylation (Fig. 5E).

Assessment of the histological sections from the liver revealed, marked reduction in lipid droplets in mice treated with sample 1, confirming the resolution of hepatic steatosis (Fig. 5F). Consistent with reductions in hepatic lipid droplet content, this intervention also reduced hepatic triglyceride content (Fig. 5G). By comparison, treatment with sample 6 did not alter hepatic triglyceride content but increased lipid droplet size, implying worsening in hepatic steatosis (Fig. 5F,G).

### Treatment of mice with myriocin containing *Cordyceps* alters the composition of gut microbiota

**Figure 6:**
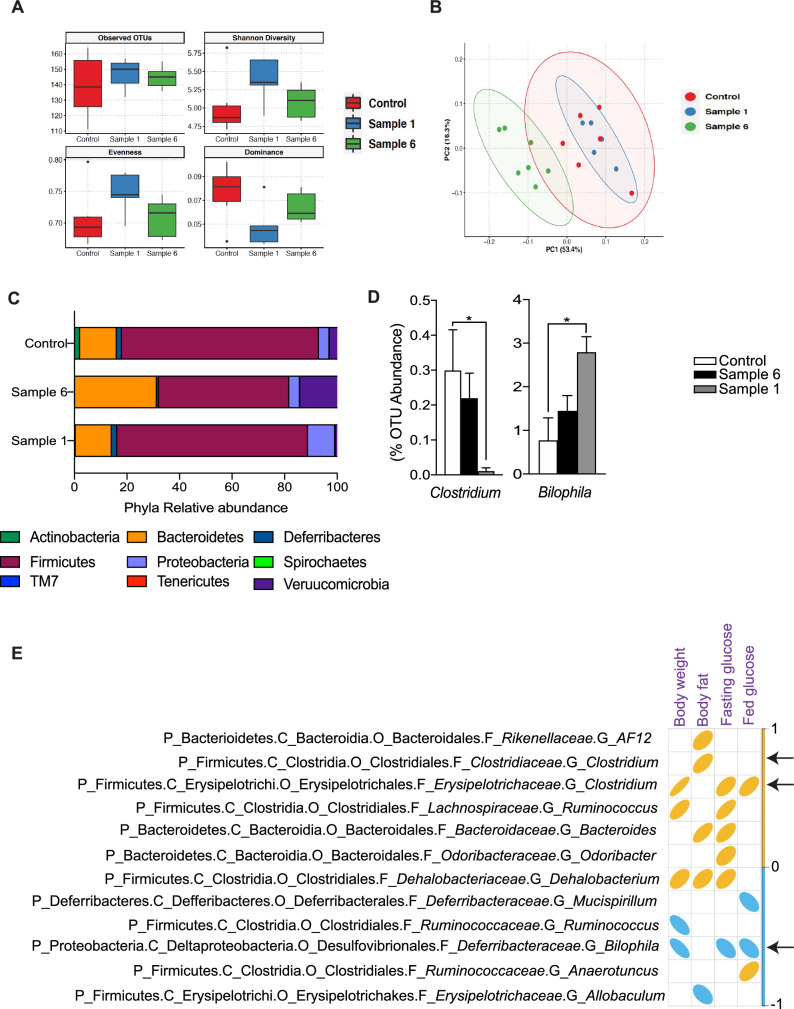
*Cordyceps* enriched in myriocin modulates gut microbial composition. C57Bl6/J mice fed high fat diets supplemented with starch (control), Sample 1 and Sample 6 for 12-weeks. Following euthanasia, cecum contents were collected and the composition of gut microbes was determined by 16S rRNA amplicon sequencing (**A**) Indices of α-diversity (Observed OTUs, Shannon Diversity, Evenness and Dominance). (**B**) β-diversity based principal component analysis (PCA) plot. (**C**) Relative abundance of gut microbes at phyla level. (**D**) Relative abundance of genera such as *Clostridium* and *Bilophila.* (**E**) Spearman’s correlation of selected gut microbes at genus level with body weight, body fat, fasting blood glucose or fed blood glucose (N = 5–7 animals per group). Values are expressed as mean ± SEM, *p < 0.05, **p < 0.001, ***p < 0.0001 vs control.

Recent studies suggest that gut microbiome could affect metabolic health and may affect insulin resistance and lipid metabolism^58^. Notably, accumulated evidence also suggests an association between dysregulated intestinal microbiome with obesity, insulin resistance and, therefore, type 2 diabetes^59^. To determine whether ceramides illicit a lipotoxic effect by modulating intestinal microbiota, we performed 16S rRNA gene sequencing to identify the composition of the gut microbiota in cecum samples isolated from animals treated with either control, Sample 1 and Sample 6. These studies revealed that although α-diversity, an indicative of species richness and evenness, was similar among the groups (Fig. 6A), the β-diversity, which represents the phylogenetic compositional differences, was significantly different at the OTU level in between the groups (Fig. 6B). Further, microbial profiling revealed significant alterations in the composition of microbes at different taxonomic levels following Sample 1 and Sample 6 treatment (Fig. 6C). Notably, Sample 1 (myriocin containing *Cordyceps*) treatment induced beneficial changes in gut microbiota as determined by a reduced abundance of *Clostriduim* genus (belong to the phyla *Fermicutes*) (Fig. 6D) which is negatively correlated with obesity^60^. Moreover, this intervention resulted in increases in the abundance of the genus *Bilophila* (belong to phyla *Proteobacteia*) (Fig. 6D) which is associated with reductions in blood glucose and improved insulin sensitivity^61,62^. In contrast, treatment with Sample 6 did not exhibit any changes in these beneficial microbes and altered the abundance in numerous microbes associated with worsening of metabolic syndrome [Bacteriodetes (*Bactereroides*, *Odoribacter*), Firmicutes (*Anaerostipes*, *Dehalobacterium and Dorea*)] despite increment in beneficial microbes such as the Verrucomicrobia (*Akkermansia*)) (Supplementary Fig. 2J).

We further performed Spearman’s correlation analysis to determine the association between the selected genera with metabolic parameters such as body weight, body fat, fasting blood glucose and fed blood glucose. Twelve specified genera were significantly associated with one or more of the metabolic parameters (Fig. 6E). In addition, eight unspecified genera belonging to the family *Coriobacteriaceae*, *Desulfovibrionaceae*, *F16*, *Mycoplasmataceae*, *Mogibacteriaceae*, *Christensenellacea* were associated with the metabolic parameters (data not shown). Notably, *Clostridium* was found to be positively associated with body weight, body fat, fasting blood glucose or fed blood glucose (Fig. 6E). Conversely, *Bilophila* was found to be negatively correlated with body weight and fasting or fed blood glucose (Fig. 6E).

## Discussion

*Cordyceps* extracts consumed as part of traditional Chinese medication regimens have previously been shown to ameliorate various diseases including metabolic anomalies^30^. However, it is not clear what is the bioactive ingredient present in *Cordyceps* extracts and what are molecular mechanisms through which these extracts elicit beneficial effects. Here we demonstrate, that presence of myriocin, a pharmacological inhibitor of ceramide biosynthesis in *Cordyceps* extract is critical for its beneficial metabolic effects. We further demonstrate that treatment of mice with *Cordyceps* extract containing myriocin at a currently recommended dose of *Cordyceps* for human consumption, reduces ceramide biosynthesis, alters gut microbial ecology and has profound effects on increasing energy expenditure, insulin sensitivity and resolving hepatic steatosis.

Ceramides accrue in obesity and recent studies from us and others suggest that these are toxic lipid metabolites that causes insulin resistance^2,7,27,50,53,63–65^. Moreover, our recent studies have suggested that ceramides are key nutrient signals that regulate energy storage and utilization^5,50^. Furthermore, inhibition of ceramide biosynthesis using myriocin in rodents improves various features of metabolic diseases such as insulin sensitivity, hepatic steatosis and cardiovascular complications^4,9,15,66^. Given the beneficial effects elicited by inhibition of ceramide biosynthesis, targeting ceramide biosynthetic pathway offers a novel strategy for the treatment of obesity and associated comorbidities. Despite this wealth of knowledge in rodents and other mammals, literature is devoid of any ceramide intervention studies that includes myriocin usage in humans. However, *Cordyceps* extracts, one of the fungi species where myriocin was originally extracted from has been used in traditional Asian medicines for centuries for multiple indications including diabetes^30^. The present study identified some of the naturally occurring or artificially grown and readily available *Cordyceps* extracts contain myriocin and suggest that a substantial number of individuals might already be consuming these myriocin enriched extracts and perhaps benefiting from the inhibition of ceramide biosynthesis. Future human intervention studies with commercially available *Cordyceps* extract such as the sample 1 are highly warranted to determine whether indeed myriocin containing *Cordyceps* extract inhibits ceramide biosynthesis and alters glucose and energy metabolism.

Systemic inhibition of ceramide biosynthesis increases energy expenditure by elevating adipose tissue thermogenic program and improves glucose uptake and metabolism by regulating Akt/PKB activation^2,3,5^. We identify *Cordyceps* extract from sample 1 indeed elicits its beneficial effects by elevating thermogenic program in adipose tissue and glucose uptake and metabolism via regulation of Akt/PKB activation (Figs. 4C, 5E). Inhibition of ceramide biosynthesis using *Cordyceps* extract from sample 1 inhibits numerous sphingolipids including, sphingomyelin, that would argue whether observed improvements are due to ceramides or another sphingolipid intermediate. Given that, ceramides but no other sphingolipids modulate thermogenic program in adipocytes and Akt/PKB activation^27,50,53^, we think these improvements were driven by exclusive inhibition of ceramides but no other sphingolipids. Surprisingly, we find that sample 6 that does not contain myriocin induces ceramide accumulation (Fig. 2B) and ensuing negative metabolic effects associated with ceramide accumulation such as decreases in energy expenditure and increases body and liver weight (Fig. 3A–J). Given that the active chemical composition in most of the commercially available *Cordyceps* preparations are not very well characterized^67^, future studies are warranted to identify active chemical compound in sample 6 that drives ceramide biosynthesis. Moreover, these data indicate that additional screening procedures should be implemented to ensure that *Cordyceps* extracts that are commercially available are safe without adverse effects.

In addition to the known mechanisms of ceramides action, the present study identifies alterations in the composition of gut microbiota following ceramide inhibition and identifies two genera *Clostridum* and *Bilophila* as the key microbes that are altered following ceramide inhibition and were found to be correlated with either body weight, fat mass and fasting blood glucose or fed blood glucose (Fig. 6D, E). Though we identified beneficial microbial changes that potentially influences systemic metabolism, we cannot rule out the possibility that these changes are secondary to a healthier gut resulting from ceramide inhibition. Therefore, future studies will seek to elucidate if these microbes contribute to some of the beneficial metabolic homeostasis attributed to ceramide inhibition.

Notably, therapeutically targeting SPT, the first enzyme in the ceramide synthetic pathway hit a road block as genetically ablating SPT presents with lethality^68^. These effects seem to be accounted by the requirement of ceramides in maintaining intestinal homeostasis^51,68^. Similarly, pharmacological inhibition of SPT, with a new class of compounds results in intestinal toxicity^52^. Despite these limitations, numerous studies have repeatedly shown robust efficacy of myriocin in inhibiting SPT activity, reducing ceramide biosynthesis and resolving various metabolic indices^6–16^. We think, the toxicity associated with SPT inhibition results from complete inhibition of SPT, as mice lacking only one allele of SPT are viable without any major developmental effects and exhibit reductions in ceramide accrual and improvements in insulin sensitivity^69^. Similarly, high dosage of *Cordyceps* containing myriocin induces intestinal toxicity compared to lower dose (Fig. 2G and data not shown). Therefore, the low dosage of *Cordyceps* containing myriocin similar to the one tested in this study, that has been consumed for centuries might provide a therapeutic opportunity to safely inhibit SPT, to reduce ceramide accumulation and improve insulin sensitivity. Moreover, purified low-dose myriocin that inhibits ceramide biosynthesis without eliciting intestinal toxicity might present an alternative strategy to inhibit ceramide biosynthesis and improve metabolic indices in humans.

Collectively, we identify that various commercially and readily available *Cordyceps* extracts currently being consumed by humans contain ceramide inhibitor myriocin and provide proof-of-principle studies that inhibition of ceramide biosynthesis with *Cordyceps* extract containing myriocin might be a readily available therapeutic option for the treatment of metabolic diseases. Moreover, given that *Cordyceps* have been thoroughly examined for not inducing mutagenicity and been assigned one of the safest supplements, these supplements might offer first-in-class ceramide intervention drugs for the treatment of metabolic diseases. However, future well-controlled studies in humans are warranted to determine if indeed *Cordyceps* treatment is safe and effectively inhibit ceramide biosynthesis and improve metabolic outcomes.

## Materials and methods

All methods were performed in accordance with relevant guidelines and regulations.

### Animal care

All animal procedures were conducted in compliance with protocols approved by the Institutional Animal Care and Use Committee (IACUC) at University of Utah and performed in accordance with the National Institute of Health *Guide for Care and Use of Laboratory Animals*. The experiments were complied with the ARRIVE guidelines^70^. C57Bl6/J male mice were purchased from Jackson Laboratories and were housed in groups of 3–5 at 22 °C–24 °C using a 12-h light/12-h dark cycle. Animals had ad libitum access to water at all times. Animals were fed a high fat diet (HFD) (D12492; Research Diets Inc., New Brunswick, NJ) supplemented with either starch or *Cordyceps* extract as stated in the manuscript from the age of 4-weeks as indicated. Euthanasia was performed using the CO_2_ inhalation a method that is consistent with the euthanasia guidelines of American Veterinarian Medical Association and approved by Institutional Animal Care and Use Committee (IACUC).

### Glucose- and insulin-tolerance tests

Glucose-tolerance tests were performed in 8-week-old mice fed with *Cordyceps* supplemented diets for 4-weeks after an overnight fast. Glucose was injected (intraperitoneal injection of a 20% solution, 10 mL/kg body weight) and blood glucose concentrations in blood were measured after 0, 15, 30, 60 and 120 min with a glucometer (Bayer Contour, Bayer, Germany). Insulin-tolerance tests were performed in 8-week-old mice fed ad libitum. After determination of basal blood glucose concentrations, each mouse received an intraperitoneal injection of insulin (0.75 IU per kg body weight; Novolin R Insulin; Novo Nordisk) and glucose concentrations in blood were measured after 15, 30, 45 and 60 min.

### Analysis of body composition

Lean and fat mass was determined via NMR™ (Bruker, Germany) in live, 12–18-week-old mice.

### Indirect calorimetry

Metabolic measurements were obtained using the CLAMS (Columbus Instruments) open-circuit indirect calorimetry system maintained at 22 °C. Food and water were provided ad libitum in the appropriate devices and measured by the built-in automated instruments. Animals were allowed to acclimatize to the cages for at least 12-h before data acquisition for additional 24-h.

### Analytical procedure

Blood glucose levels were determined from whole venous blood using an automatic glucose monitor (Bayer Contour, Bayer, Germany). Insulin levels in serum were measured by Cisbio Insulin assay using mouse standards according to manufacturer’s guidelines (Cisbio).

### Myriocin extraction from *Cordyceps*

Myriocin was extracted from *Cordyceps* using a similar protocol described before^71^. Briefly, extraction of myriocin was performed by weighing 0.5 g of dried *Cordyceps* powder which was then sonicated with 95% methanol (MeOH) (3 × 10 mL) for 40 min at 40 °C. The extract solutions were combined and centrifuged at 10,000 rpm/min for 10 min then evaporated under vacuum. For liquid samples 5 and 7, 10 mL of the liquid extracts were directly used for evaporation. The residue was dissolved in 2 mL of 50% methanol and centrifuged at 12,000 rpm/min for 10 min. The supernatant was then loaded onto an Oasis HLB 3 cc solid-phase extraction (SPE) column (Waters Corp, Milford, MA) pretreated with 10 mL of 100% MeOH, followed by 5 mL of H_2_O. After sample loading, the column was washed with 10% MeOH (1 × 3 mL) and 50% MeOH (1 × 3 mL) successively, and then the myriocin-containing fraction was eluted with 80% methanol (2 × 3 mL). The eluent was evaporated under a gentle stream of nitrogen in a water bath at 50 °C. The residue was then resuspended in 250 µL of 80% MeOH and centrifuged at 12,000 r/min for 10 min. The supernatant was then transferred to a glass vial for LC–MS analysis. Myriocin (M1177, Sigma, St. Louis, MO, USA) was suspended in MeOH to a final concentration of 1 μM to be used as positive control.

### LC–MS analysis of myriocin content in *Cordyceps*

*Cordyceps* extracts were separated on a Poroshell 120 EC-C18 column (2.1 × 150 mm; 1.9 µm) (Agilent Technologies, Santa Clara, CA, USA) maintained at 35 °C connected to an Agilent HiP 1290 Sampler, Agilent 1290 Infinity pump, and Agilent 6545 Accurate Mass Q-TOF dual AJS-ESI mass spectrometer operated in negative ion mode. Source gas temperature was set to 250 °C, with a gas (N2) flow of 12 L/min and a nebulizer pressure of 35 psi. Sheath gas temperature was 325 °C, sheath gas (N2) flow of 11 L/min, capillary voltage is 3500 V, and nozzle voltage 0 V. Mobile phase A consisted of acetonitrile (ACN):water (5:95 v/v), mobile phase B consisted of ACN:water (95:5 v/v), and both contained 0.1% formic acid. The chromatography gradient started at 10% mobile phase B, holds for 1 min, increases to 100% B from 1 to 10 min, where it is held until 12 min and then returned to starting conditions. Post-time was 3 min and the flowrate was 0.4 mL/min throughout. Injection volume was 10 µL and the samples were analyzed in a randomized order. Myriocin was monitored using accurate mass *m/z* 400.2705 of [M − H]^−^ with the retention time established by the standard, and quantitated based on the ratio to the myriocin standard injected separately.

### Lipidomics

Lipid extracts were separated on an Acquity UPLC CSH C18 1.7 µm 2.1 × 50 mm column maintained at 60 °C connected to an Agilent HiP 1290 Sampler, Agilent 1290 Infinity pump, and Agilent 6490 triple quadrupole (QqQ) mass spectrometer. Sphingolipids were detected using dynamic multiple reaction monitoring (dMRM) in positive ion mode. Source gas temperature was set to 210 °C, with a gas (N_2_) flow of 11 L/min and a nebulizer pressure of 30 psi. Sheath gas temperature was 400 °C, sheath gas (N_2_) flow of 12 L/min, capillary voltage is 4000 V, nozzle voltage 500 V, high pressure RF 190 V and low-pressure RF was 120 V. Injection volume was 2 µL and the samples were analyzed in a randomized order with the pooled QC sample injection eight times throughout the sample queue. Mobile phase A consisted of ACN: H_2_O (60:40 v/v) in 10 mM ammonium formate and 0.1% formic acid, and mobile phase B consisted of IPA: ACN:H_2_O (90:9:1 v/v) in 10 mM ammonium formate and 0.1% formic acid. The chromatography gradient was started at 15% mobile phase B, increased to 30% B over 1 min, increased to 60% B from 1 to 2 min, increased to 80% B from 2 to 10 min, and increased to 99% B from 10 to 10.2 min where it was held until 14 min. Post-time was 5 min and the flowrate was 0.35 mL/min throughout. Collision energies and cell accelerator voltages were optimized using sphingolipid standards with dMRM transitions as [M + H]^+^ → [*m/z* = 284.3] for dihydroceramides, [M + H]^+^ → [*m/z* = 264.2], for ceramides, [M-H_2_O + H]^+^ → [*m/z* = 271.3] for d7-isotope labeled ceramides and [M + H]^+^ → [*m/z* = 184.1] for sphingomyelins. Sphingolipids without available standards were identified based on HR-LC/MS, quasi-molecular ion and characteristic product ions. Their retention times were either taken from HR-LC/MS data or inferred from the available sphingolipid standards. Results from LC–MS experiments were collected using Agilent Mass Hunter Workstation and analyzed using the software package Agilent Mass Hunter Quant B.07.00. Sphingolipids were quantitated based on peak area ratios to the standards added to the extracts.

### Determining the composition of gut microbiota using 16s rRNA amplicon sequencing

Microbial profiling was carried out using established protocols in identifying the composition of gut microbiota following dietary treatment^72^. Cecal contents were used to extract genomic DNA using the DNeasy PowerSoil Kit (Qiagen, Md, USA). Fifty nanograms of genomic DNA were used for the amplification of the V4 variable region of the 16S rRNA gene using the 515F/806R primers. Library preparation was performed using the Nextera XT DNA Library Preparation Kit (Illumina, Cat# FC-131-1096) and individual barcodes were labelled with Nextera XT indices to accommodate multiplexing. The Illumina MiSeq platform with of ~ 30% PhiX DNA was used for pooled amplicons, paired-end sequencing (2 × 250 bp)^73^. Standardized pipelines in QIIME 2 were used for the microbial ecology matrices^74^. Alpha diversity metrics for richness, diversity and evenness at the phylum, genus and OTU-levels were determined in QIIME using the observed features, Shannon diversity index and Pielou’s evenness index, respectively. Bray–Curtis dissimilarities were used to evaluate beta diversity and to conduct the principal coordinate analysis (PCoA). Spearman’s correlations were performed using the Shiny App between bacterial abundance data, body weight, body fat, fasting blood glucose and fed blood glucose were used to determine the associations between the abundance of specific bacterial species and metabolic parameters^75^. All data were considered statistically significant at p < 0.05, and are expressed as mean ± SEM, where appropriate.

### Histology and immunochemistry

For histology, tissues were fixed in 4% formalin, embedded in paraffin, sectioned at 5 μm and stained with hematoxylin and eosin or immunostained with antibodies directed against UCP-1 (abcam: ab10983). Intestinal lesions were evaluated by a board-certified veterinary pathology using post-examination masking to group assignment^76^. Quantification of cell size was performed with Image J software (NIH).

### Western blot analysis

Proteins were extracted from tissues or cultured cells by homogenizing or scraping in RIPA buffer (0.5% NP-40, 0.1% sodium deoxycholate, 150 mM NaCl, 50 mM Tris–HCl, pH 7.5) containing protease inhibitors (Complete Mini, Roche). The homogenate was cleared by centrifugation at 4 °C for 30 min at 15,000*g* and the supernatant containing the protein fraction recovered. Protein concentration in the supernatant was determined using the BCA Protein Assay Kit (Pierce). 20 µg of proteins was resolved using precast Bolt-PAGE (4–12% Bis–Tris gels, Invitrogen) and transferred to Nitrocellulose membranes (GE Healthcare). Membranes were blocked with 5% BSA in Tris-buffered saline containing 0.2% Tween-20 (TBS-T) and incubated with primary antibodies at 4 °C overnight.

### Statistics

Data were plotted as the mean ± SEM. Student *t*-test, one-way or two-way ANOVA were carried out using Prism (GraphPad Prism) and statistical significance was considered meaningful at p < 0.05.

## Supplementary Information

Supplementary Figures.

## References

[1] Chaurasia B, Summers SA (2015). Ceramides—Lipotoxic inducers of metabolic disorders. Trends Endocrinol. Metab..

[2] Chaurasia B, Summers SA (2020). Ceramides in metabolism: Key lipotoxic players. Annu. Rev. Physiol..

[3] Li Y, Talbot CL, Chaurasia B (2020). Ceramides in adipose tissue. Front. Endocrinol. (Lausanne).

[4] Chaurasia B, Talbot CL, Summers SA (2020). Adipocyte ceramides-the Nexus of inflammation and metabolic disease. Front. Immunol..

[5] Summers SA, Chaurasia B, Holland WL (2019). Metabolic Messengers: ceramides. Nat. Metab..

[6] Holland WL, Summers SA (2008). Sphingolipids, insulin resistance, and metabolic disease: New insights from in vivo manipulation of sphingolipid metabolism. Endocr. Rev..

[7] Holland WL (2007). Inhibition of ceramide synthesis ameliorates glucocorticoid-, saturated-fat-, and obesity-induced insulin resistance. Cell Metab..

[8] Bikman BT (2012). Fenretinide prevents lipid-induced insulin resistance by blocking ceramide biosynthesis. J. Biol. Chem..

[9] Ussher JR (2010). Inhibition of de novo ceramide synthesis reverses diet-induced insulin resistance and enhances whole-body oxygen consumption. Diabetes.

[10] Hojjati MR (2005). Effect of myriocin on plasma sphingolipid metabolism and atherosclerosis in apoE-deficient mice. J. Biol. Chem..

[11] Hojjati MR, Li Z, Jiang XC (2005). Serine palmitoyl-CoA transferase (SPT) deficiency and sphingolipid levels in mice. Biochim. Biophys. Acta.

[12] Jiang XC, Goldberg IJ, Park TS (2011). Sphingolipids and cardiovascular diseases: Lipoprotein metabolism, atherosclerosis and cardiomyopathy. Adv. Exp. Med. Biol..

[13] Park TS, Rosebury W, Kindt EK, Kowala MC, Panek RL (2008). Serine palmitoyltransferase inhibitor myriocin induces the regression of atherosclerotic plaques in hyperlipidemic ApoE-deficient mice. Pharmacol. Res..

[14] Russo SB (2012). Ceramide synthase 5 mediates lipid-induced autophagy and hypertrophy in cardiomyocytes. J. Clin. Investig..

[15] Yang G (2009). Central role of ceramide biosynthesis in body weight regulation, energy metabolism, and the metabolic syndrome. Am. J. Physiol. Endocrinol. Metab..

[16] Park TS (2008). Ceramide is a cardiotoxin in lipotoxic cardiomyopathy. J. Lipid Res..

[17] Poss AM (2019). Machine learning reveals serum sphingolipids as cholesterol-independent biomarkers of coronary artery disease. J. Clin. Investig..

[18] Hilvo M (2019). Development and validation of a ceramide- and phospholipid-based cardiovascular risk estimation score for coronary artery disease patients. Eur. Heart J..

[19] Wigger L (2017). Plasma dihydroceramides are diabetes susceptibility biomarker candidates in mice and humans. Cell Rep..

[20] Apostolopoulou M (2018). Specific hepatic sphingolipids relate to insulin resistance, oxidative stress, and inflammation in nonalcoholic steatohepatitis. Diabetes Care.

[21] Meeusen JW (2018). Plasma ceramides. Arterioscler. Thromb. Vasc. Biol..

[22] Vasile VC (2021). Ceramide scores predict cardiovascular risk in the community. Arterioscler. Thromb. Vasc. Biol..

[23] Anroedh S (2018). Plasma concentrations of molecular lipid species predict long-term clinical outcome in coronary artery disease patients. J. Lipid Res..

[24] Havulinna AS (2016). Circulating ceramides predict cardiovascular outcomes in the population-based FINRISK 2002 cohort. Arterioscler. Thromb. Vasc. Biol..

[25] Laaksonen R (2016). Plasma ceramides predict cardiovascular death in patients with stable coronary artery disease and acute coronary syndromes beyond LDL-cholesterol. Eur. Heart J..

[26] Mantovani A (2018). Association of plasma ceramides with myocardial perfusion in patients with coronary artery disease undergoing stress myocardial perfusion scintigraphy. Arterioscler. Thromb. Vasc. Biol..

[27] Chaurasia B (2016). Adipocyte ceramides regulate subcutaneous adipose browning, inflammation, and metabolism. Cell Metab..

[28] Yu J, Xu H, Mo Z, Zhu H, Mao X (2009). Determination of myriocin in natural and cultured Cordyceps cicadae using 9-fluorenylmethyl chloroformate derivatization and high-performance liquid chromatography with UV-detection. Anal. Sci..

[29] Wang S (2009). Simultaneous determination of nucleosides, myriocin, and carbohydrates in Cordyceps by HPLC coupled with diode array detection and evaporative light scattering detection. J. Sep. Sci..

[30] Olatunji OJ (2018). The genus Cordyceps: An extensive review of its traditional uses, phytochemistry and pharmacology. Fitoterapia.

[31] Panda AK, Swain KC (2011). Traditional uses and medicinal potential of *Cordyceps sinensis* of Sikkim. J. Ayurveda Integr. Med..

[32] Zhu JS, Halpern GM, Jones K (1998). The scientific rediscovery of a precious ancient Chinese herbal regimen: *Cordyceps sinensis*: Part II. J. Altern. Complement Med..

[33] Zhu JS, Halpern GM, Jones K (1998). The scientific rediscovery of an ancient Chinese herbal medicine: *Cordyceps sinensis*: Part I. J. Altern. Complement Med..

[34] Abdel-Fatah TM (2014). Clinicopathological significance of human apurinic/apyrimidinic endonuclease 1 (APE1) expression in oestrogen-receptor-positive breast cancer. Breast Cancer Res. Treat..

[35] Peng J (2013). Anti-fibrotic effect of Cordyceps sinensis polysaccharide: Inhibiting HSC activation, TGF-beta1/Smad signalling, MMPs and TIMPs. Exp. Biol. Med. (Maywood).

[36] Paterson RR (2008). Cordyceps: A traditional Chinese medicine and another fungal therapeutic biofactory?. Phytochemistry.

[37] Lo HC, Hsu TH, Tu ST, Lin KC (2006). Anti-hyperglycemic activity of natural and fermented *Cordyceps sinensis* in rats with diabetes induced by nicotinamide and streptozotocin. Am. J. Chin. Med..

[38] Lo HC, Tu ST, Lin KC, Lin SC (2004). The anti-hyperglycemic activity of the fruiting body of Cordyceps in diabetic rats induced by nicotinamide and streptozotocin. Life Sci..

[39] Balon TW, Jasman AP, Zhu JS (2002). A fermentation product of *Cordyceps sinensis* increases whole-body insulin sensitivity in rats. J. Altern. Complement Med..

[40] Ahn MY, Jee SD, Lee BM (2007). Antiobesity effects of *Isaria sinclairii* by repeated oral treatment in obese Zucker rats over a 4-month period. J. Toxicol. Environ. Health A.

[41] Kiho T, Yamane A, Hui J, Usui S, Ukai S (1996). Polysaccharides in fungi. XXXVI. Hypoglycemic activity of a polysaccharide (CS-F30) from the cultural mycelium of *Cordyceps sinensis* and its effect on glucose metabolism in mouse liver. Biol. Pharm. Bull..

[42] Bao ZD, Wu ZG, Zheng F (1994). Amelioration of aminoglycoside nephrotoxicity by *Cordyceps sinensis* in old patients. Zhongguo Zhong Xi Yi Jie He Za Zhi.

[43] Lu L (2002). Study on effect of *Cordyceps sinensis* and artemisinin in preventing recurrence of lupus nephritis. Zhongguo Zhong Xi Yi Jie He Za Zhi.

[44] Ahn MY (2004). Genotoxicity evaluation of *Isaria sinclairii* (ISE) extract. J. Toxicol. Environ. Health A.

[45] Vasiljevic JD (2016). *Cordyceps sinensis*: Genotoxic potential in human peripheral blood cells and antigenotoxic properties against hydrogen peroxide by comet assay. Altern. Ther. Health Med..

[46] McGuffin M, Hobbs C, Upton R, Goldberg A (1997). American Herbal Products Association’s Botanical Safety Handbook.

[47] Lin, B. & Li, S. Cordyceps as an herbal drug. In *Herbal Medicine: Biomolecular and Clinical Aspects* (eds. Benzie, I.F.F. & Wachtel-Galor, S.) (2011). 22593937

[48] Chen W, Zhang W, Shen W, Wang K (2010). Effects of the acid polysaccharide fraction isolated from a cultivated *Cordyceps sinensis* on macrophages in vitro. Cell Immunol..

[49] Dong CH, Yao YJ (2008). In vitro evaluation of antioxidant activities of aqueous extracts from natural and cultured mycelia of *Cordyceps sinensis*. Lebensm Wiss Technol..

[50] Chaurasia B (2019). Targeting a ceramide double bond improves insulin resistance and hepatic steatosis. Science.

[51] Li Z (2018). Sphingolipid de novo biosynthesis is essential for intestine cell survival and barrier function. Cell Death Dis.

[52] Genin MJ (2016). Imidazopyridine and pyrazolopiperidine derivatives as novel inhibitors of serine palmitoyl transferase. J. Med. Chem..

[53] Chaurasia B (2020). Ceramides are necessary and sufficient for diet-induced impairment of thermogenic adipocytes. Mol. Metab..

[54] Summers SA, Garza LA, Zhou H, Birnbaum MJ (1998). Regulation of insulin-stimulated glucose transporter GLUT4 translocation and Akt kinase activity by ceramide. Mol. Cell Biol..

[55] Powell DJ, Turban S, Gray A, Hajduch E, Hundal HS (2004). Intracellular ceramide synthesis and protein kinase Czeta activation play an essential role in palmitate-induced insulin resistance in rat L6 skeletal muscle cells. Biochem. J..

[56] Blouin CM (2010). Plasma membrane subdomain compartmentalization contributes to distinct mechanisms of ceramide action on insulin signaling. Diabetes.

[57] Stratford S, Hoehn KL, Liu F, Summers SA (2004). Regulation of insulin action by ceramide: dual mechanisms linking ceramide accumulation to the inhibition of Akt/protein kinase B. J. Biol. Chem..

[58] Gomez-Arango LF (2016). Connections between the gut microbiome and metabolic hormones in early pregnancy in overweight and obese women. Diabetes.

[59] Gerard C, Vidal H (2019). Impact of gut microbiota on host glycemic control. Front. Endocrinol. (Lausanne).

[60] Peters BA (2018). A taxonomic signature of obesity in a large study of American adults. Sci. Rep..

[61] Moreno-Indias I (2016). Insulin resistance is associated with specific gut microbiota in appendix samples from morbidly obese patients. Am. J. Transl. Res..

[62] Lecomte V (2015). Changes in gut microbiota in rats fed a high fat diet correlate with obesity-associated metabolic parameters. PLoS One.

[63] Turpin SM (2014). Obesity-induced CerS6-dependent C16:0 ceramide production promotes weight gain and glucose intolerance. Cell Metab..

[64] Hammerschmidt P (2019). CerS6-derived sphingolipids interact with Mff and promote mitochondrial fragmentation in obesity. Cell.

[65] Xia JY (2015). Targeted induction of ceramide degradation leads to improved systemic metabolism and reduced hepatic steatosis. Cell Metab..

[66] Ji, R.*, et al.* Increased de novo ceramide synthesis and accumulation in failing myocardium. *JCI Insight.* **2 **, 1–19. 10.1172/jci.insight.82922 (2017). 10.1172/jci.insight.82922 PMC5414571 28469091

[67] Hsu TH, Shiao LH, Hsieh CY, Chang DM (2002). A comparison of the chemical composition and bioactive ingredients of the Chinese medicinal mushroom DongChongXiaCao, its counterfeit and mimic, and fermented mycelium of Cordyceps sinensis. Food Chem..

[68] Ohta E (2009). Analysis of development of lesions in mice with serine palmitoyltransferase (SPT) deficiency-Sptlc2 conditional knockout mice. Exp. Anim..

[69] Li Z (2011). Reducing plasma membrane sphingomyelin increases insulin sensitivity. Mol. Cell Biol..

[70] Percie du Sert N (2020). The ARRIVE guidelines 2.0: Updated guidelines for reporting animal research. BMJ Open Sci..

[71] Cheng WM (2017). Identification and determination of myriocin in *Isaria cicadae* and its allies by LTQ-Orbitrap-HRMS. Mycology.

[72] Petersen C (2019). Dietary supplementation with strawberry induces marked changes in the composition and functional potential of the gut microbiome in diabetic mice. J. Nutr. Biochem..

[73] Wankhade UD (2017). Enhanced offspring predisposition to steatohepatitis with maternal high-fat diet is associated with epigenetic and microbiome alterations. PLoS One.

[74] Bolyen E (2019). Reproducible, interactive, scalable and extensible microbiome data science using QIIME 2. Nat. Biotechnol..

[75] Piccolo BD (2018). Dynamic assessment of microbial ecology (DAME): A web app for interactive analysis and visualization of microbial sequencing data. Bioinformatics.

[76] Meyerholz DK, Beck AP (2018). Principles and approaches for reproducible scoring of tissue stains in research. Lab. Investig..

