# Supplementary material for: Cordyceps inhibits ceramide biosynthesis and improves insulin resistance and hepatic steatosis
Source: Sci Rep. 2022 May 4;12:7273. doi: 10.1038/s41598-022-11219-3 (PMC9068713; doi:10.1038/s41598-022-11219-3)

Cordyceps inhibits ceramide biosynthesis and improves insulin resistance and hepatic steatosis

Ying Li, Chad Lamar Talbot, Bhawna Chandravanshi, Alec Ksiazek, Ayushi Sood, Kamrul H Chowdhury, J. Alan Maschek, James Cox, Adhini Kuppuswamy Satheesh Babu, Henry A. Paz, Pon Velayutham Anandh Babu, David K Meyerholz, Umesh D Wankhade, William Holland, E Shyong Tai, Scott A Summers, Bhagirath Chaurasia

Supplementary Figure Legends

Figure S1. Screening of *Cordyceps* extracts for myriocin content via mass-spectrometry. Related to Figure 1. (A) LC/MS chromatogram of purified myriocin and *Cordyceps* Sample 2 (B), Sample 3 (C), Sample 4 (D), Sample 5 (E), Sample 7 (F) and Sample 8 (G) depicting myriocin content.

Figure S2. Characterization of C57Bl6/J mice fed high fat diets supplemented with *Cordyceps*. Related to Figure 3 and 4. Following 4-weeks (A-D) and 12-weeks (E-H) of dietary intervention supplemented with *Cordyceps* animals were placed in metabolic cages (CLAMS) from Columbus Instruments. During the subsequent 3-days, (A and E) Food intake, (B and F) Respiratory exchange ratio (RER), (C-D and G-H) ambulatory activity was quantified (N=4-7 animals per group). (I) Following euthanasia, serum insulin content was determined (N=5 animals per group). C57Bl6/J mice fed high fat diets supplemented with starch (control), Sample 1 and Sample 6 for 12-weeks. Following euthanasia, cecum content was collected and microbial content was determined by 16S sequencing. (J) Relative abundance of genera modulated by *Cordyceps* supplementation. Values are expressed as mean ± SEM, *$p<0.05$, **$p<0.001$, ***$p<0.0001$ vs control.

Ying et al Supplementary Figure 1

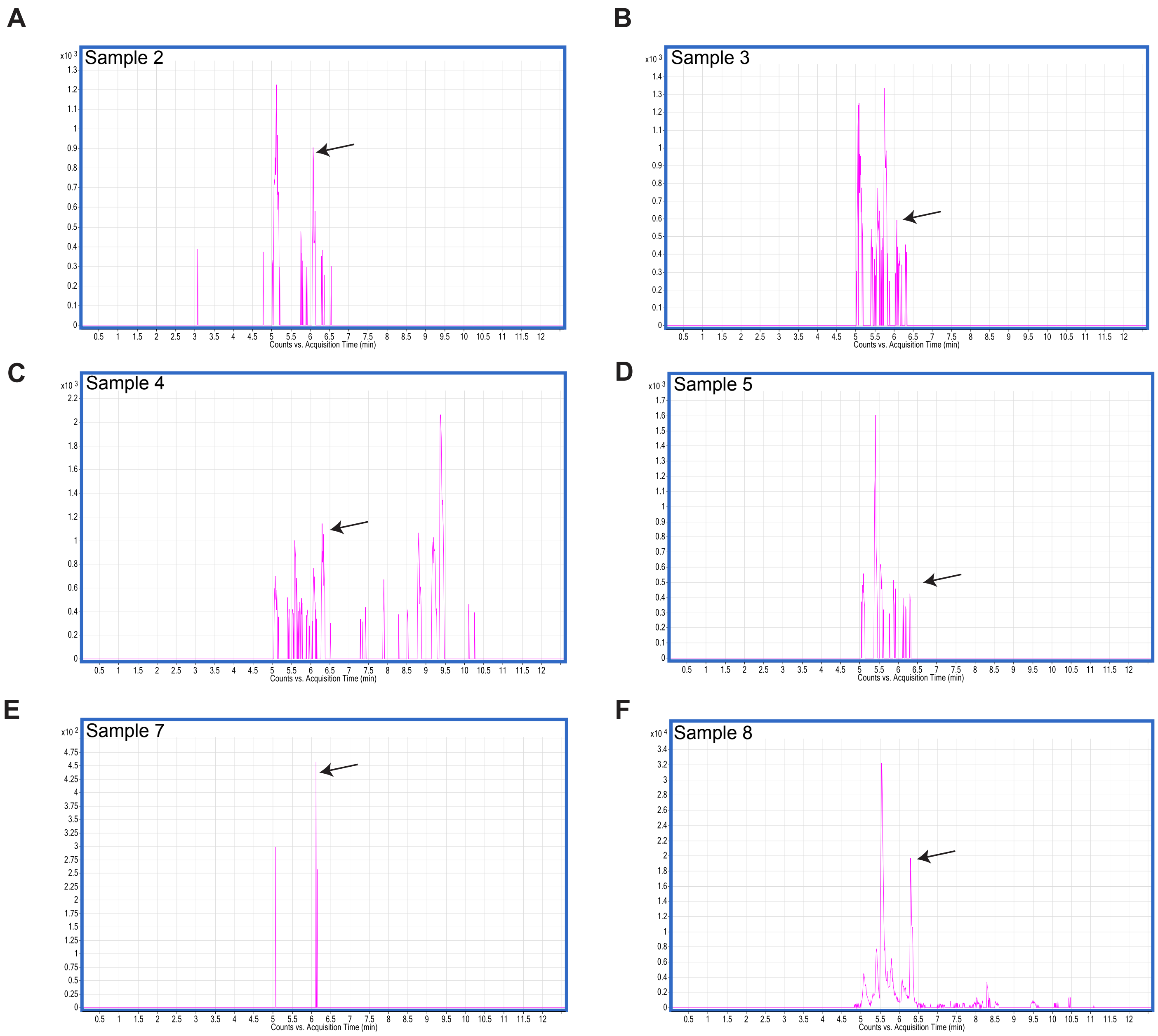

Ying et al Supplementary Figure 2

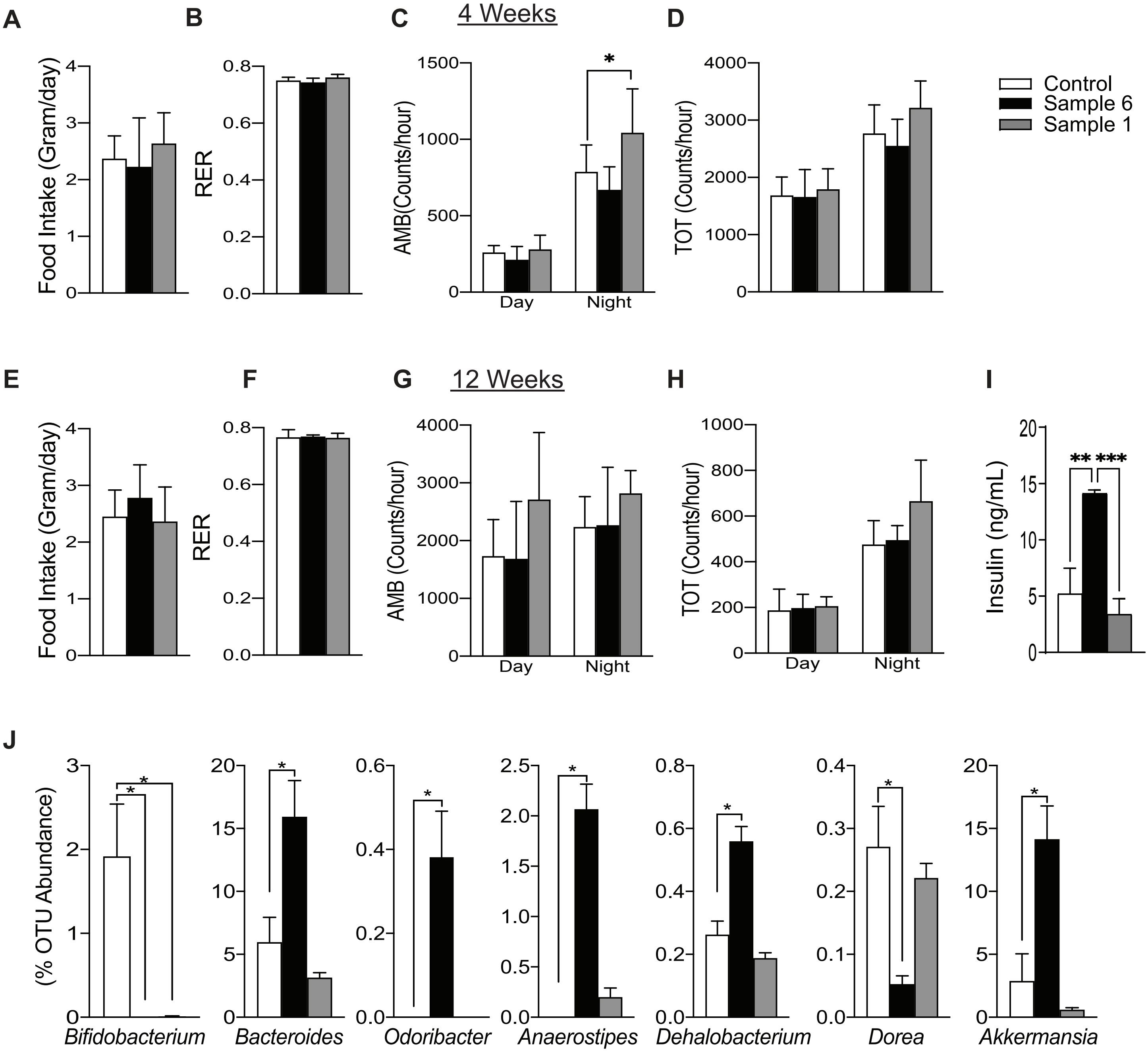

Ying et al Unprocessed images related to Figure 5E

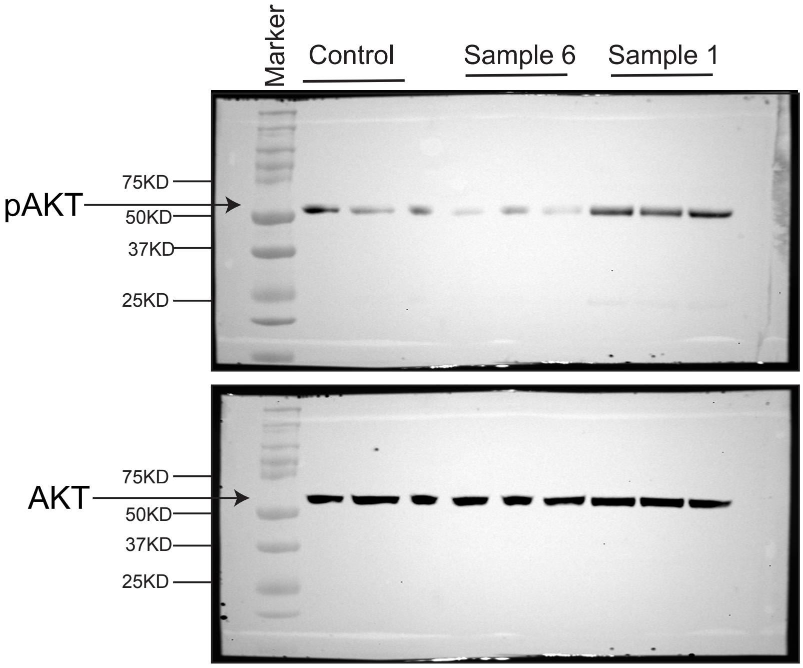

Supplement: Supplementary file 1 — Supplementary Figures. [file 41598_2022_11219_MOESM1_ESM.pdf]
